# Prohibitin Is Involved in Patients with IgG4 Related Disease

**DOI:** 10.1371/journal.pone.0125331

**Published:** 2015-05-01

**Authors:** Hongwu Du, Lili Shi, Peng Chen, Weikang Yang, Yiping Xun, Chunhe Yang, Lanqing Zhao, Yabin Zhou, Guangyu Chen

**Affiliations:** 1 112 Lab, School of Chemistry and Biological Engineering, University of Science and Technology Beijing, Beijing, China; 2 ImmunoHunt Corporation, Beijing, China; Institute of Immunology, Rikshospitalet, NORWAY

## Abstract

**Objective:**

IgG4-related disease (IgG4-RD) is a chronic systemic disease involved in many organs and tissues. As only limited autoantigens have been found since the beginning of this century, the aim of this study was to reveal new candidate autoantigens of IgG4-RD.

**Methods:**

Multiple cell lines including HT-29, EA.hy926, HEK 293 and HepG2 were used to test the binding ability of circulating autoantibodies from IgG4-RD sera. The amino-acid sequence was then analyzed by matrix-assisted laser desorption/ionization time-of-flight tandem (MALDI-TOF/TOF) mass spectrometry. After the cloning and expression of recombinant putative autoantigen in a bacterial expression system, the corresponding immuno assay was set up and utilized to observe the prevalence of serum autoantibodies in a large set of confirmed clinical samples.

**Results:**

One positive autoantigen was identified as prohibitin. ELISA analysis showed that a majority of patients with IgG4-RD have antibodies against prohibitin. Anti-prohibitin antibodies were present in the sera of patients with definite autoimmune pancreatitis (25/34; 73.5%), Mikulicz’s disease (8/15; 53.3%), retroperitoneal fibrosis (6/11; 54.5%), other probable IgG4-RD (26/29; 89.7%) and Sjögren’s syndrome (4/30; 13.3%) but not in apparently healthy donors (1/70; 1.4%).

**Conclusions:**

An association between prohibitin and patients with some IgG4-RD was observed, although the results were quite heterogeneous among different individuals within autoimmune pancreatitis, Mikulicz’s disease and retroperitoneal fibrosis.

## Introduction

IgG4-RD has recently been recognized as a distinct clinical entity comprising a number of disorders, such as type 1 autoimmune pancreatitis (AIP) [1,2], sclerosing cholangitis [3], Mikulicz’s disease (MD) [4], tubulointerstitial nephritis [5] and retroperitoneal fibrosis (RPF) [6] etc. It is a chronic systemic disease with multi-organ involvement and IgG4-positive plasma cell infiltration [7]. The characteristics of IgG4-RD are high density of serum IgG4 and osmotic IgG4-positive plasma cells, which can infiltrate pancreas [8], salivary [9], lacrimal glands [10], lung [11], retroperitoneal space [6], kidneys [12], pituitary [13], eyes [14] and others [15]. Due to lack of specific clinical features in the early stage, current clinical practice greatly relies on the examination of histology, imaging and serology, which increases the difficulty of a clear diagnosis. Autoimmunity has been considered the most probable pathogenesis of IgG4-RD [16] and several autoantigens have been suggested, including lactoferrin [17], carbonic anhydrase II (CAII) [18], amylase-alpha 2A [19], pancreatic secretory trypsin inhibitor (PSTI) [20] and plasminogen-binding protein peptide [21], with autoantibodies against these targets successfully examined in patients with IgG4-RD in laboratories.

Furthermore, the molecular mechanism of IgG4-RD is not fully clarified yet, and it requires the discovery of more associated molecules through additional research. It is hoped that some of these molecules will be targeted in clinical applications, whereas others may be used to study the disease pathogenesis and how IgG4-RD relates to other autoimmune diseases. The aim of this study was to screen and identify new autoantigens which are closely related to the morbidity of IgG4-RD.

## Materials and Methods

### Samples

In this study, sera from 89 patients with IgG4-RD with an average age of 38 years (range 21 to 68, 36 female and 53 male) who conform to the criteria proposed by the Japan Pancreas Society [22], sera from 30 patients with Sjögren’s syndrome (SjS) (35 years old: range 15 to 58, 21 female and 9 male) who were diagnosed according to the standard defined criteria [23], and sera from 70 matching healthy donors (36 years old: range 15 to 60, 46 female and 24 male) were enrolled as control. This study was approved by the ethical committee of the Peking Union Medical College Hospital, and each patient involved in this study provided written informed consent. Furthermore, written informed consent on behalf of the minors involved in the study was obtained from their guardians. Blood specimens were procured and medical record information including gender, age, height, weight and supplement use was collected. Sera were isolated, aliquoted and stored at -80°C until used.

### Cell lines

HT-29, EA.hy926, HEK 293 and HepG2 cell lines were all purchased from American Type Culture Collection (Rockville, MD). To cultivate the four cell lines above, DMEM (HyClone, UT) with 10% fetal bovine serum (HyClone, UT) was used as the culture medium. Because IgG4-RD can occur in various organs, which increases the complexity of the disease, HT-29 was selected as the representative of glandular epithelial cells; EA.hy926 as the representative of vein endothelial cells; and HEK 293 and HepG2 as the representatives of human organs involvement.

### Indirect immunofluorescence assays

Indirect immunofluorescence assays were performed as previously described [24]. HT-29, EA.hy926, HEK 293 and HepG2 cell lines were plated on the slides. The cells were then cultured on the slides overnight, fixed with 4% paraformaldehyde and followed by incubation with TritonX-100. Sera from patients with IgG4-RD, SjS or healthy donors were diluted at 1: 20 in PBS, and incubated with the slides for 2 hours at 37°C. After extensive washing, the slides were incubated with fluorescein isothiocyanate (FITC)-conjugated goat-anti-human IgG (Bioss, Beijing, China) which had been diluted 1: 150 in PBS for 30 min. The slides were then examined under a fluorescence microscope (AMG, Bothell, WA). For the indirect immunofluorescence assay, the total cell fluorescence ratio was obtained by Image J software (NIH, MD).

### HT-29 cell-based ELISA

HT-29 cells were plated in 96-well plates and cultured at 37°C. Cells were fixed with 4% paraformaldehyde and then washed with 5 ‰ PBST (Tween-20 in PBS, v/v). After fixation with 0.2% TritonX-100 for 10 min, cells were washed by using 5 ‰ PBST again, and allowed to dry. Then sera were diluted at 1: 100 with PBS and incubated with the cells. After incubation, horseradish peroxidase-conjugated goat anti-human IgG at a dilution of 1: 10000 (ImmunoHunt, Beijing, China) was added. The binding of antibody was quantified by the addition of tetramethylbenzidine (TMB, 1 mg/mL, Sigma). The chromogenic reaction was stopped with 2 M H_2_SO_4_ and the plates were obtained spectrophotometrically at 450 nm on a microplate reader (Tecan, Hombrechtikon, Switzerland).

### Western blotting

The detailed procedure of Western blotting was performed as described elsewhere [25]. In brief, to screen the autoantibodies in the sera, HT-29 cells were lysed in RIPA buffer (Beyotime, Shanghai, China) with 1% protease inhibitor cocktail (Sigma-Aldrich, MO), combined with the loading buffer and boiled for 10 min. After removing the insoluble fraction by centrifuge, the eluted proteins were resolved by 12% SDS-PAGE and then transferred onto PVDF membrane (Merck Millipore, MA). Next the membrane was cut into 2-cm wide stripes. After blocking with PBS containing 5% nonfat milk for 1 hour at 37°C, the membrane was incubated with sera at a dilution of 1: 500. Horseradish peroxidase-conjugated goat anti-human IgG was used as the secondary antibody with a dilution of 1: 10,000 for 1 hour at 37°C. The positive bands were detected with enhanced chemiluminescence kit (Applygen, Beijing, China).

### Immunoprecipitation

Total extracts of HT-29 cells (500 μg) were incubated with 9 μL mixed sera (equal volumes from three positive IgG4-RD patients in western blotting detection) overnight at 4°C on a rotator. Subsequently, 50 μL of protein A-Sepharose beads (Sigma, MO) washed with PBS were added and incubated for 4 hours at 4°C. The immunoprecipitates were washed three times in 200 μL PBS. Then, the immunoprecipitates were suspended in a sample loading buffer and analyzed by 12% SDS-PAGE.

### In-gel digestion and mass spectrometry analysis

In-gel digestion and MALDI-TOF/TOF MS analysis were performed as previously described [24]. The gel band of interest was excised and detained by 25 mM NH_4_HCO_3_, containing 50% acetonitrile and dried with vacuum centrifugation. Then, 25 mM NH_4_HCO_3_ including 10 mM dithiotreitol (DTT) was added to cover the gel pieces and allowed to reduce for 2 hours at 37°C. After cooling to room temperature, the DTT solution was replaced by the same volume 25 mM NH_4_HCO_3_ containing 55 mM iodoacetamide to incubate for 45 min in the dark. After the gel pieces were washed and completely dried in a speed-vac, they were swollen in a digestion buffer containing 50 mM NH_4_HCO_3_ and 12.5 ng/μL of trypsin in an ice-cold bath. After 1 hour ice-cold bath, the supernatant was removed and replaced with 50 mM NH_4_HCO_3_ to keep the gel pieces wet during enzymatic cleavage. MALDI-TOF/TOF MS analysis of the tryptic digestion solution was performed on 4700 Proteomics Analyzer mass spectrometer (Applied, Biosystems). CHCA (α-cyano-4-hydroxycinnamic acid) matrix was prepared by dissolving 5 mg in 1 mL of 50:50 acetonitrile/water containing 0.1% tallow fatty acid. The mass spectrometric data were analyzed using the Mascot bioinformatics database search engine (Matrix Sciences, London, UK). Peptide sequences were interpreted from the MS/MS spectra by searching the Homo sapiens subset of the Swissprot protein database. Search parameters including carbamidomethylation of cysteines were set as a fixed modification, and then methionine oxidation was set as a variable modification, respectively. The peptide mass tolerance was set at ±100 ppm and the fragment mass tolerance at ±0.6 Da. Trypsin was specified as the proteolytic enzyme, and one missed cleavage was allowed.

### Expression and purification of target antigen

The total RNA was isolated from HT-29 using TRIzol reagent (Invitrogen, CA). RT-PCR was performed according to the manufacturer’s instructions (Fermentas, MD). Human PHB protein was over-expressed in the *E*.*coli* BL21, followed by purification with Ni-NTA resin (Qiagen, Hilden, Germany). Protein concentrations were determined by BCA kit (Boisynthesis Biotechnology, Beijing, China). Purified recombinant protein was stored at -80°C.

### Human recombinant PHB based-ELISA

ELISAs were performed as described previously [25]. Briefly, 0.1 μg/mL of recombinant protein was used to coat 96-well microtiter plates overnight at 4°C. After blocking with goat serum, sera from patients or controls were diluted at 1: 100 and added to the antigen-coated wells and incubated for 2 hours. Bound antibodies were incubated with horseradish peroxidase-conjugated mouse anti-human IgG4 (Abcam, MA) which had been diluted at 1:4,000 and reacted with TMB as substrate. Finally the reaction was stopped by adding 2 M H_2_SO_4_. The absorbance at 450 nm was measured with a microplate reader (Tecan, Hombrechtikon, Switzerland).

### Statistical analysis

To determine whether the frequency of autoantibodies in each cohort of patients’ sera was significantly higher than that in sera from healthy donor, the statistical analysis was evaluated by SPSS software (Version 17, Chicago, IL), with results considered to be statistically significant if *P*< 0.05. The critical point for positive definition was the number with a higher value than that of the healthy donors (Mean + 3 SD).

## Results

### HT-29 was selected as target for the antigen screening

**Fig 1:**
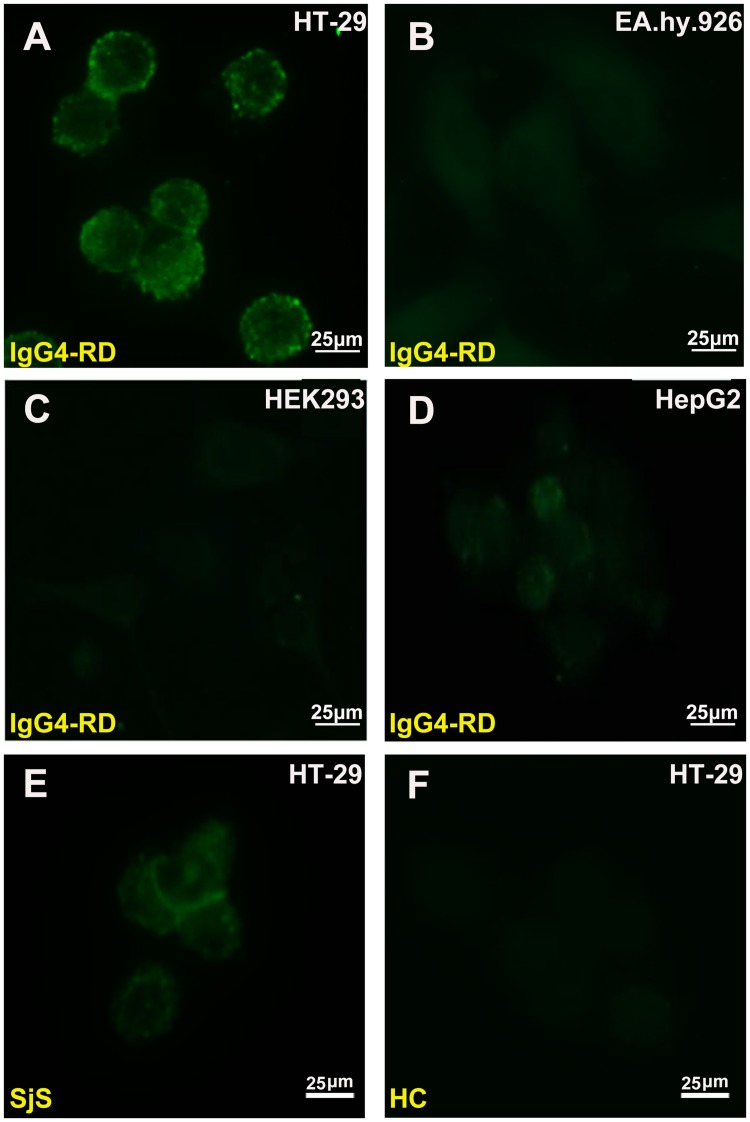
Immunofluorescence analysis. (A-D) Immunofluorescence was performed on HT-29 by confocal laser microscopy, then compared with other cells including EA.hy926, HEK 293 and HepG2. Total cell fluorescence was analyzed by Image J software and significant differences were found between HT-29 and three other cell lines (*p*<0.0001), indicating positive reactions in HT-29 cells with IgG4-RD sera. (E-F) Control samples including SjS and HC were then tested on HT-29 cells, and the sera of patients with SjS were found to have a weaker specific reaction and no positive signal on HC.

The autoantibody profile of IgG4-RD in HT-29, EA.hy926, HEK 293 and HepG2 cell lines was tested (Fig 1A–1D). The existences of autoantigens in the HT-29 cells were demonstrated by the presence of abundant fluorescence. Concurrently, by using different control sera to incubate with HT-29 cells, it was shown that the sera from SjS and HC have a relatively weaker or non-specific interaction (Fig 1E–1F).

### Antibody titer

**Fig 2:**
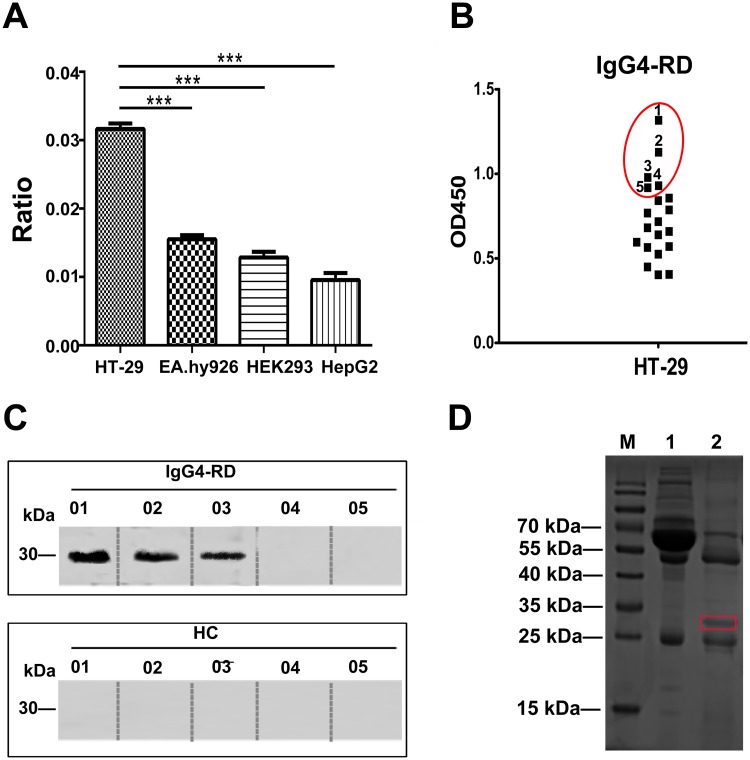
Identification of target antigen. (A) The value of cell fluorescence was analyzed by Image J software. *** indicates *P*<0.0001. (B) Five of 20 patients with IgG4-RD presenting relatively higher optical density values on ELISA for HT-29 cell were selected for Western blotting. (C) Western blotting of HT-29 cell extracts with the sera from 5 IgG4-RD patients showed a positive band with a molecular weight of approximately 30 kDa in 3 patients but not in healthy controls. (D) Immunoprecipitation was performed by incubating the extracts of HT-29 with IgG4-RD patient sera and an approximately 30 kDa protein band reacted with antibodies from IgG4-RD patients.

Total fluorescent differences among four different cells lines were quantified by Image J software and significant differences were observed (Fig 2A). ELISA of HT-29 with the sera from 20 IgG4-RD patients (the first batch of samples in the lab) was performed. The sera from 5 IgG4-RD patients which presented relatively higher optical density in ELISA were selected to perform the subsequent Western blotting experiment (Fig 2B).

### PHB was identified as a target protein in IgG4-RD patients

Through Western blotting analysis, a specific positive band near 30 kDa of molecular weight was observed from the reaction between 3 IgG4-RD sera and HT-29 cells (Fig 2C). However, no positive reaction was observed with healthy controls.

Immunoprecipitation was further carried out by incubating the extract of HT-29 cells with mixed sera, which resulted in an approximately 30 kDa protein band (Fig 2D). This protein was found to share approximately 40% sequence similarity with human PHB (NCBI accession number, gi|4505773; Mascot score, 692), and 8 unique peptides of PHB were matched.

### Expression and purification of recombinant human PHB

**Fig 3:**
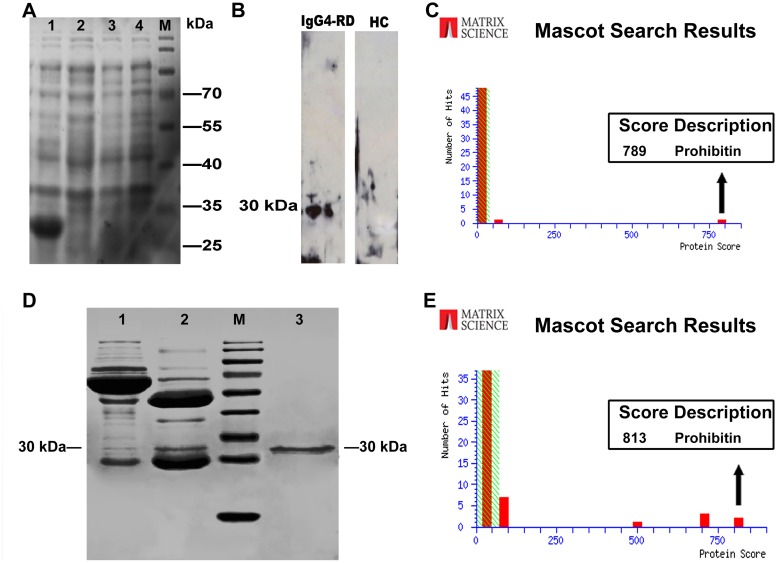
Verification of prohibitin. (A) The cloning, expression and purification of recombinant PHB protein. M, protein markers; lane 1, cell extracts of pET-28a (+)-PHB/BL21 after IPTG induction for 6 hour at 37°C; lane 2, cell extracts of pET-28a (+)-PHB/BL21 before IPTG induction; lane 3, cell extracts of pET-28a (+)-BL21 after IPTG induction. lane 4, cell extracts of BL21 after IPTG induction. (B) Western blot using purified PHB protein showed that only the sera from patients with IgG4-RD (lane 1) rather than HC (lane 2) contain antibodies against a 30 kDa cellular protein. (C) The expressed protein was purified and further identified by MS, which revealed its identity as PHB. (D) PHB protein was also identified in immunoprecipitates; lane 1, supernatant of immunoprecipitation; lane 2, immunoprecipitates; lane 3, control sample (the purified rhPHB). (E) The protein band on lane 2 was excised and identified by MALDI-TOF/TOF MS, which again revealed its identity as PHB.

The recombinant expression plasmid pET-28a (+)-PHB was transformed into *E*.*coli* BL21 (DE). After 6 hours of induction by isopropyl-b-D-thiogalactopyranoside, the cells were lysed by ultrasonic and the extracts were analyzed by SDS-PAGE. High levels of expression of recombinant PHB protein were observed (Fig 3A).

### Western blotting and immunoprecipitation

To further demonstrate the immunoreactivity of PHB protein against the patients’ sera, purified PHB was analyzed with western blotting using mixed sera. It was shown that the sera from patients with IgG4-RD but not from healthy donors could recognize the recombinant PHB protein. (Fig 3B).

After the purification of recombinant PHB protein with Ni-NTA resin, the eluted fractions were separated on SDS-PAGE and further confirmed by mass spectrometry (Fig 3C). Moreover, immunoprecipitation was further employed to confirm whether the PHB protein was a real IgG4-RD autoantigen. It was shown that the band of PHB protein was clearly present in the immunoprecipitates, further indicating that the PHB protein was a target of IgG4-RD (Fig 3D). The protein bands were excised and also identified by mass spectrometry (Fig 3E).

### The prevalence of anti-PHB antibodies in IgG4-RD patients

**Fig 4:**
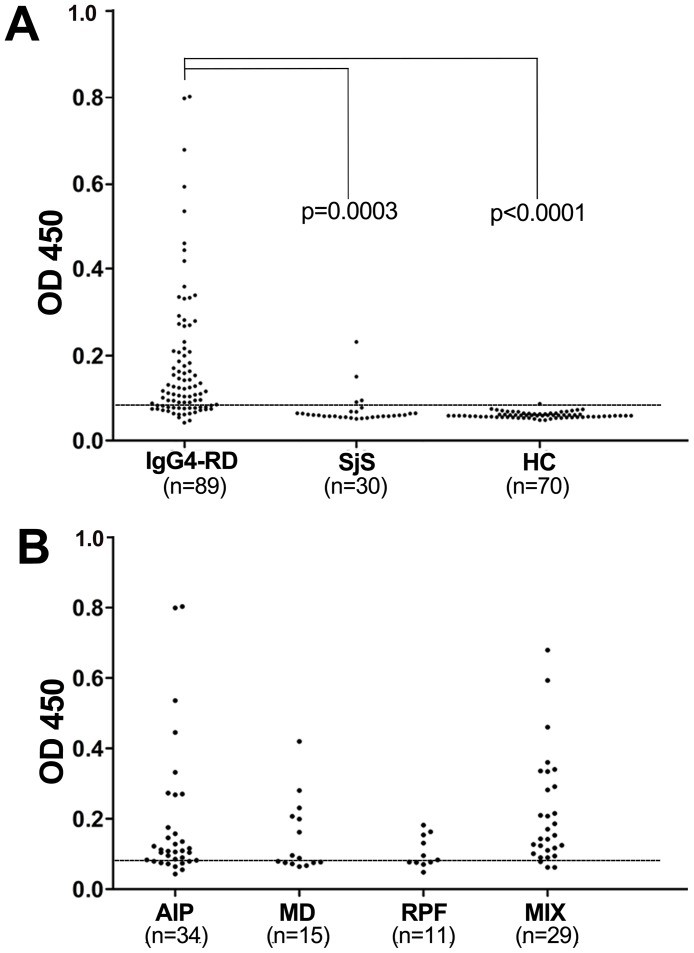
Anti-PHB autoantibodies induced in IgG4-RD patients. (A) The prevalence of autoantibodies against human PHB in sera from patients was observed. ELISA was used to detect the reactivity of serum IgG4 against recombinant human PHB protein. The anti-PHB antibodies were detected in 65 of 89 RA patients (73%), 4 of 30 SjS patients (13.3%) and 1 of 70 healthy donors (1.4%). The reactivity of anti-PHB antibodies was significantly higher than HC (***P<0.0001). (B) The patients with IgG4-RD were then divided into the following confirmed subtypes: AIP, definite autoimmune pancreatitis (25/34, 73.5%); MD, Mikulicz’s disease (8/15, 53.3%); RPF, retroperitoneal fibrosis (6/11, 54.5%); MIX, affect multiply organs (26/29, 89.7%).

The prevalence of autoantibodies against human PHB was examined in various patients by ELISA. A total of 189 samples were tested in this study. Of the 89 sera with IgG4-RD analyzed, 65 (73%) were found to be reactive with PHB, but only 1 (1.4%) of the 70 healthy donors was positive (Fig 4A). Anti-PHB antibodies were present in the sera of patients with a different subtype of IgG4-RD (Fig 4B) including definite AIP (25/34; 73.5%), MD (8/15; 53.3%), RPF (6/11; 54.5%), and other probable IgG4-RD (26/29; 89.7%).

## Discussion and Conclusions

One advantage of this study is that the indirect immunofluorescence analysis for multiple cell lines was performed at the beginning to screen out enrichment of candidate autoantigen targets. Actually, dozens of cell lines of different origin were used in the pre-screening process (data not shown). The fluorescence signal intensity indicated the binding ability of autoantigens with the sera from IgG4-RD, in which the promising cell lines with clear positive signals were selected and used for the next screening circle for putative target autoantigens. After the in-gel digestion and mass spectrometry analysis, the potential autoantigen was revealed to be PHB.

PHB is involved in the developing process of many diseases [26] and may act as a tumor suppressor that can activate anti-proliferative activity by inhibiting cell cycle and the synthesis of DNA. Using ELISA, the levels of anti-PHB antibodies were clearly higher in sera from IgG4-RD than in healthy controls, leading us to hypothesize that the existence of anti-PHB autoantibodies can accelerate cell proliferation which can originally inhibit the biological function of PHB, resulting in tissue enlargement. Moreover, MHC binding prediction tool from IEDB Analysis Resource was used to determine potential MHC-I binding motif in PHB. A consensus result by both Artificial neural network (ANN) and Stabilized matrix method (SMM) indicated peptide VSDDLTERA (from Val_150_ to Gla_158_) with low IC50 (nM) being a good MHC-I binder [27–31].

By investigating rat models of unilateral ureteral obstruction and renal tubule interstitial fibrosis, researchers discovered that the expression of PHB was significantly lower in renal tissues, leading them to assume that PHB may play a role in interstitial fibrosis [32]. Besides, Low expression of PHB has also been observed in inflammatory bowel disease and may help to reduce the pain of colonic inflammation [33]. It has been hypothesized that PHB may be related to innate immunity and may participate in the mediation of immune function and inflammation. However, more research is needed to clarify the exact role that PHB plays in IgG4-RD.

In summary, this study has revealed that PHB is associated with some IgG4-RD patients, including those in different IgG4-RD subtypes such as AIP, MD, and RPF. However, the pathology role and clinical value of PHB remains unclear and need further investigations.

## References

[1] Deshpande V, Chicano S, Finkelberg D, Selig MK, Mino-Kenudson M, Brugge WR, et al Autoimmune pancreatitis: a systemic immune complex mediated disease. Am J Surg Pathol. 2006;30: 1537–1545. 17122509 10.1097/01.pas.0000213331.09864.2c

[2] Okazaki K, Uchida K, Miyoshi H, Ikeura T, Takaoka M, Nishio A. Recent concepts of autoimmune pancreatitis and IgG4-related disease. Clin Rev Allerg Immu. 2011;41: 126–138. 10.1007/s12016-010-8214-2 21170607

[3] Kawaguchi K, Koike M, Tsuruta K, Okamoto A, Tabata I, Fujita N. Lymphoplasmacytic sclerosing pancreatitis with cholangitis: a variant of primary sclerosing cholangitis extensively involving pancreas. Hum Pathol. 1991;22: 387–395. 2050373 10.1016/0046-8177(91)90087-6

[4] Masaki Y, Kurose N, Umehara H. IgG4-related disease: a novel lymphoproliferative disorder discovered and established in Japan in the 21st century. J Clin Exp Hematopathol. 2011;51: 13–20. 21628856 10.3960/jslrt.51.13

[5] Saeki T, Nishi S, Imai N, Ito T, Yamazaki H, Kawano M, et al Clinicopathological characteristics of patients with IgG4-related tubulointerstitial nephritis. Kidney Int. 2010;78: 1016–1023. 10.1038/ki.2010.271 20720530

[6] Hamanou H, Kawa S, Ochi Y, Unno H, Shiba N, Wajiki M, et al Hydronephrosis associated with retroperitoneal fibrosis and sclerosing pancreatitis. Lancet. 2002;359: 1403–1404. 11978339 10.1016/s0140-6736(02)08359-9

[7] Guma M, Firestein GS. IgG4-related diseases. Best Pract Res Clin Rheumatol. 2012;26: 425–438. 10.1016/j.berh.2012.07.001 23040358

[8] Yoshida K, Toki F, Takeuchi T, Watanabe S, Shiratori K, Hayashi N. Chronic pancreatitis caused by an autoimmune abnormality. Digest Dis Sci. 1995;40: 1561–1568. 7628283 10.1007/BF02285209

[9] Gill J, Angelo N, Yeong ML, McIvor N. Salivary duct carcinoma arising in IgG4-related autoimmune disease of the parotid gland. Hum Pathol. 2009;40: 881–886. 10.1016/j.humpath.2008.10.020 19200575

[10] Mikulicz JV. Concerning a peculiar symmetrical disease of the lacrimal and salivary glands. Modern Classics 1938: 165–186.

[11] Zen Y, Kitagawa S, Minato H, Kurumaya H, Katayanagi K, Masuda S, et al IgG4-positive plasma cells in inflammatory pseudotumor (plasma cell granuloma) of the lung. Hum Pathol. 2005;36: 710–717. 16084938 10.1016/j.humpath.2005.05.011

[12] Cornell LD, Chicano SL, Deshpande V, Collins AB, Selig MK, Lauwers GY, et al Pseudotumors due to IgG4 immune-complex tubulointerstitial nephritis associated with autoimmune pancreatocentric disease. Am J Surg Pathol. 2007;31: 1586–1597. 17895762 10.1097/PAS.0b013e318059b87c

[13] Stone JH. IgG4-related disease: nomenclature, clinical features, and treatment. Semin Diagn Pathol. 2012;29: 177–190. 10.1053/j.semdp.2012.08.002 23068296

[14] Wallace ZS, Khosroshahi A, Jakobiec FA, Deshpande V, Hatton MP, Ritter J, et al IgG4-related systemic disease as a cause of “idiopathic” orbital inflammation, including orbital myositis, and trigeminal nerve involvement. Surv Ophthalmol. 2012;57: 26–33. 10.1016/j.survophthal.2011.07.004 22018678

[15] Khosroshahi A, Stone JH. A clinical overview of IgG4-related systemic disease. Curr Opin Rheumatol. 2011;23: 57–66. 10.1097/BOR.0b013e3283418057 21124086

[16] Zen Y, Nakanuma Y. Pathogenesis of IgG4-related disease. Curr Opin Rheumatol. 2011;23: 114–118. 10.1097/BOR.0b013e3283412f4a 21045701

[17] Okazaki K, Uchida K, Ohana M, Nakase H, Uose S, Inai M, et al Autoimmune-related pancreatitis is associated with autoantibodies and a Th1/Th2-type cellular immune response. Gastroenterology. 2000;118: 573–581. 10702209 10.1016/s0016-5085(00)70264-2

[18] Nishi H, Tojo A, Onozato ML, Jimbo R, Nangaku M, Uozaki H, et al Anti-carbonic anhydrase II antibody in autoimmune pancreatitis and tubulointerstitial nephritis. Nephrol Dial Transpl. 2007;22: 1273–1275. 17138573 10.1093/ndt/gfl672

[19] Endo T, Takizawa S, Tanaka S, Takahashi M, Fujii H, Kamisawa T, et al Amylase alpha-2A autoantibodies: novel marker of autoimmune pancreatitis and fulminant type 1 diabetes. Diabetes. 2009;58: 732–737. 10.2337/db08-0493 19001184 PMC2646073

[20] Asada M, Nishio A, Uchida K, Kido M, Ueno S, Uza N, et al Identification of a novel autoantibody against pancreatic secretory trypsin inhibitor in patients with autoimmune pancreatitis. Pancreas. 2006;33: 20–26. 16804408 10.1097/01.mpa.0000226881.48204.fd

[21] Frulloni L, Lunardi C, Simone R, Dolcino M, Scattolini C, Falconi M, et al Identification of a novel antibody associated with autoimmune pancreatitis. New Engl J Med. 2009;361: 2135–2142. 10.1056/NEJMoa0903068 19940298

[22] Umehara H, Okazaki K, Masaki Y, Kawano M, Yamamoto M, Saeki T, et al Comprehensive diagnostic criteria for IgG4-related disease (IgG4-RD), 2011. Mod Rheumatol. 2012;22: 21–30. 10.1007/s10165-011-0571-z 22218969

[23] Homma M, Tojo T, Akizuki M, Yamagata H. Criteria for Sjogren's syndrome in Japan. Scand J Rheumatol. 1985;61: 26–27. 3473640

[24] Xun Y, Chen P, Yan H, Yang W, Shi L, Chen G, et al Identification of prohibitin as an antigen in Behcet's disease. Biochem Bioph Res Co. 2014;451:389–393. 10.1016/j.bbrc.2014.07.126 25091478

[25] Du H, Li C, Jin H, Chen G, Xun Y. Generation and evaluation of antibodies against human MGF E-peptide by reverse phase protein microarray and reverse competitive ELISA. Bioanalysis. 2013;5: 2269–2275. 10.4155/bio.13.195 24053242

[26] Theiss AL, Sitaraman SV. The role and therapeutic potential of prohibitin in disease. Biochim Biophys Acta. 2011;1813: 1137–1143. 10.1016/j.bbamcr.2011.01.033 21296110 PMC3370678

[27] Kim Y, Ponomarenko J, Zhu Z, Tamang D, Wang P, Greenbaum J, et al Immune epitope database analysis resource. Nucleic Acids Res. 2012;40: W525–530. 10.1093/nar/gks438 22610854 PMC3394288

[28] Nielsen M, Lundegaard C, Worning P, Lauemøller SL, Lamberth K, Buus S, et al Reliable prediction of T-cell epitopes using neural networks with novel sequence representations. Protein Sci. 2003;12: 1007–1017. 12717023 10.1110/ps.0239403 PMC2323871

[29] Lundegarrd C, Lamberth K, Harndahl M, Buus S, Lund O, Nielsen M. NetMHC-3.0: Accurate web accessible predictions of Human, Mouse, and Monkey MHC class I affinities for peptides of length 8–11. Nucleic Acids Res. 2008;36: W509–512. 10.1093/nar/gkn202 18463140 PMC2447772

[30] Peters B, Sette A. Generating quantitative models describing the sequence specificity of biological processes with the stabilized matrix method. BMC Bioinformatics. 2005;6: 132 15927070 10.1186/1471-2105-6-132 PMC1173087

[31] Sidney J, Assarsson E, Moore C, Ngo S, Pinilla C, Sette A, et al Quantitative peptide binding motifs for 19 human and mouse MHC class I molecules derived using positional scanning combinatorial peptide libraries. Immunome Res. 2008;4: 2 10.1186/1745-7580-4-2 18221540 PMC2248166

[32] Zhou TB, Qin YH, Zhou C, Lei FY, Zhao YJ, Chen J, et al Less expression of prohibitin is associated with increased Caspase-3 expression and cell apoptosis in renal interstitial fibrosis rats. Nephrology. 2012;17: 189–196. 10.1111/j.1440-1797.2011.01522.x 21914039

[33] Mishra S, Murphy LC, Nyomba BL, Murphy LJ. Prohibitin: a potential target for new therapeutics. Trends Mol Med. 2005;11: 192–197. 15823758 10.1016/j.molmed.2005.02.004

